# Waterfall Forest Environment Regulates Chronic Stress via the NOX4/ROS/NF-κB Signaling Pathway

**DOI:** 10.3389/fneur.2021.619728

**Published:** 2021-03-18

**Authors:** Zixin Zhu, Xueke Zhao, Qiuyue OuYang, Yinghui Wang, Yan Xiong, Shuo Cong, Mingyu Zhou, Manman Zhang, Xinhua Luo, Mingliang Cheng

**Affiliations:** ^1^Department of Pathophysiology, Guizhou Medical University, Guiyang, China; ^2^Department of Infectious Diseases, Affiliated Hospital of Guizhou Medical University, Guiyang, China; ^3^Department of Clinical Laboratory, Affiliated Hospital of Guizhou Medical University, Guiyang, China; ^4^Department of Infectious Diseases, Guizhou Provincial People's Hospital, Guiyang, China; ^5^Department of Gastroenterology, Guizhou Provincial People's Hospital, Guiyang, China

**Keywords:** chronic fatigue, waterfall forest environment, chronic stress, NOX4/ROS/NF-κB signaling pathway, immunity

## Abstract

**Background:** Forest therapy has been proven to have beneficial effects on people with depression and anxiety. However, it remains unknown whether the waterfall forest environment (WF) affects the physical and psychological health of patients with chronic fatigue and how the WF regulates chronic stress.

**Methods:** Twenty-four patients with chronic fatigue were randomly divided into two groups: the WF group and the urban (U) group. Scores on the Hamilton Anxiety Scale (HAMA), Hamilton Depression Scale (HAMD), and Fatigue Scale-14 (FS-14) were evaluated before and after environmental intervention. Detection of physiological indexes and inflammatory factor levels and immunological analysis were also performed. In addition, the chronic stress rat model was constructed, and the effects of the WF on hopelessness and liver damage of rats were investigated.

**Results:** Patients with chronic fatigue in the WF group showed a significant decrease in FS-14, HAMA, and HAMD scores compared with the U group. The expression levels of glutathione peroxidase and superoxide dismutase were remarkably higher in the WF group than in the U group. However, the expression levels of malondialdehyde and inflammatory factors (IL-1β, TNF-α, IL-6, and IL-10) were remarkably decreased after the intervention of the WF. In addition, animal experiments confirmed that the WF improved hopelessness, liver damage, and excitability of neurons of chronic stress rats. Mechanistically, the WF reduced the liver damage caused by chronic stress in rats by inhibiting the NOX4/ROS/NF-κB signaling pathway.

**Conclusions:** Collectively, the WF had a positive effect on immune enhancement and physical and psychological health in patients with chronic fatigue and might inhibit chronic stress by regulating the NOX4/ROS/NF-κB signaling pathway.

## Background

Chronic fatigue syndrome (CFS) is a disorder characterized by prolonged fatigue, sore throat, muscle pain, joint pain, weakness, sleep disturbance, memory loss, and poor concentration (1). The pathogenesis of CFS is complicated and barely understood. A variety of causes result in the development of CFS. It has been demonstrated that neuroendocrine, environmental factors, bioenergy metabolisms, immune system disabilities, and hormonal imbalances in the hypothalamic-pituitary-adrenal axis play primary roles (2, 3). Recently, clinical treatments mainly include symptomatic therapies, drugs, and exercise therapy. These treatments can alleviate symptoms and improve life quality (4). However, the effectiveness of these treatments is limited. Therefore, it is necessary to find a new and effective method for treating CFS.

It is well-known that the natural environment plays an important role in promoting human health. Recently, forest therapy attracts widespread attention and gradually emerges in Japan and Korea (5, 6). Studies have reported that forest therapy could remarkably improve adults' mental health by reducing stress, anxiety, and depression symptoms (7, 8). In addition, forest therapy significantly enhanced parasympathetic nerve activity and inhibited sympathetic nerve activity compared with the urban environment (9). Furthermore, forest therapy trips enhanced natural killer cell activity and increased immune function (10). Due to the positive physical and psychological effects of forest therapy, it is used as an intervention method for treating many diseases including high blood pressure, diabetes, and depression. Bielinis et al. discovered that forest therapy had a positive effect on the mental health of patients with affective and psychotic disorders (11). However, little is known about the effect of forest therapy on patients with chronic fatigue.

In addition to the forest environment, the waterfall environment has also been found to have a variety of beneficial health effects (12). The waterfall is characterized by negatively charged and inhalable nano-water aerosols, which are considered to trigger numerous biological effects (13). Increasing evidence suggested that negative air ions in the waterfall had a positive effect on immunity and physical symptoms by interacting with phytoncides released from trees (14). The waterfall environment was reported to improve lung function and psychological stress parameters (depression, anxiety, and paranoid ideation) in people with moderate to high stress levels (15). In addition, Gaisberger et al. discovered that the waterfall environment reduced inflammatory responses and improved asthma symptoms and lung function in patients with allergic asthma (12).

In many cases, the natural environment promotes human health by immune regulation. High-altitude climate therapy has been found to regulate the TH2/Treg cell ratio and the levels of IL-10 (16). A previous study reported that activated immune-inflammatory and oxidative pathways played a vital role in the pathogenesis of CFS (17). Cytokines including tumor necrosis factor-α (TNF-α), IL-6, and IL-10 were considered as key biomarkers for CFS (18). In addition, reactive oxygen species (ROS) are common in many biological systems. ROS are involved in various signaling pathways including physiological and pathophysiological stresses (19). Studies have reported that the nicotinamide adenine dinucleotide phosphate oxidase (NOX) enzymatic family, especially NOX4, is one of the main sources of ROS (20). Simultaneously, the NF-κB signaling pathway is a downstream pathway of ROS and can regulate inflammation and oxidative stress. Dai et al. discovered that isoquercetin can ameliorate oxidative stress and neuronal apoptosis after ischemia/reperfusion injury via inhibiting the NOX4/ROS/NF-κB signaling pathway (21). Therefore, we speculated that the waterfall forest environment (WF) can regulate oxidative stress via the NOX4/ROS/NF-κB signaling pathway.

In this study, we investigated the effects of the WF on psychology and physiology of patients with chronic fatigue. In addition, we constructed a chronic stress rat model and further explored the effects of the WF on chronic stress via the NOX4/ROS/NF-κB signaling pathway. This study would provide the novel therapeutic approach for chronic fatigue and give us deep insight into the mechanism by which the WF regulated chronic stress.

## Methods

### Participants

Twenty-four patients with chronic fatigue (9 men, 39.4 ± 10.7 years of age, BMI 21.9 ± 2.1; 15 women, 42.3 ± 8.7 years of age, BMI 22.1 ± 1.8) were enrolled in this study. Patients were recruited through Internet and poster advertisements. Chronic fatigue is an intermediate state between physiological fatigue and pathological fatigue. Therefore, to determine chronic fatigue, we need exclude physiological fatigue and organic diseases and mental diseases (such as depression). Participants' perception and psychological changes were evaluated before enrollment by professional psychologists. The diagnosis of chronic fatigue according to the revised chronic fatigue criteria of the U.S. Centers for Disease Control Diagnostic criteria includes (1) Main Symptoms: fatigue lasting 6 months or more based on excluding other ailments and (2) meeting at least four of the following symptoms: (1) short-term memory loss or inability to concentrate; (2) sore throat; (3) swollen lymph nodes; (4) muscular soreness; (5) joint pain without redness and swelling; (6) headache; (7) inability to recover energy after sleep; (8) physical discomfort for 24 h after physical or mental work; (9) lack of sleep; (10) discomfort after fatigue; and (11) cognitive problems and/or orthostatic intolerance.

Meanwhile, in addition to meeting the diagnosis of chronic fatigue, the following inclusion and exclusion criteria were followed when screening patients for this study.

Inclusion criteria:

1. People (20–50 years of age) with moderate or heavy work in urban areas.
2. BMI index between 19 and 25.
3. People who have recently presented with mental tiredness, depression, insomnia, forgetfulness, decreased sleep quality, and decreased concentration.
4. Patients with or without lymph nodes with mild swelling and pain in the neck and armpit, but having normal physical examination and routine laboratory tests.

Exclusion criteria:

1. Women preparing for pregnancy or pregnant and lactating women.
2. Patients with mental diseases, hypothyroidism, or diabetes and requiring long-term medication and regular follow-up visits.
3. Patients who have recently (within 6 months) undergone anesthetic surgery.
4. Patients who depend on drugs, alcohol, cocaine, cannabis, or amphetamines.

Exit criteria:

1. The participants could not adapt to the changes in the environment and have poor appetite, and their sleep quality is significantly reduced. They are eager to improve by quitting the experiment.
2. The participants developed severe acute lesions (respiratory infection and digestive inflammation) during the course of the study and need medical treatment or hospitalization.
3. The participants had accident injuries (car accident, fracture, cold, etc.) or important family emergencies (death, marriage, divorce, etc.).

All participants obtained informed consent. This study was approved by the Ethics Committee of Guizhou Provincial People's Hospital.

### Study Site

The study in the experimental control group was performed in Guiyang, Guizhou province, China (106°17′E 26°11′N). The study in the experimental group was conducted in the core scenic area of Huangguoshu Waterfall, Anshun city, Guizhou province, China (150° 40′E 25°59.5′N).

### Study Design

The experiment was conducted from August 19 to 25, 2019. All participants were divided into 2 groups, including the WF group (*n* = 12) and the urban (U) group (*n* = 12) by a computer-generated randomization code. The control variables for the participants are as follows:

1. Alcohol consumption and smoking were restricted 24 h before and throughout the experiment.
2. Food intake is limited 8 h before blood drawing in each experiment.
3. Treatment regimens involving other natural environments have not been administered.
4. Excessive activity is limited outside the test area.
5. Excessive use of electronic mobile devices (more than 8 h/day) or staying up late is limited.
6. A regular diet provided throughout the study.

The WF group went to Huangguoshu Waterfall for natural environment intervention. The participants in the WF group lived in the hotel near the core scenic area of Huangguoshu Waterfall (~500 m) and performed daily activity in the WF for at least 3 h. The U group was in the routine working environment. Participants in the U group took regular work and performed activity in the green landscape area of Guiyang city for at least 3 h during the rest. All subjects wore uniform sports bracelets during this period. Daily sleep time and activity intensity were measured to ensure that there were no statistically significant differences in these values. Before the beginning of the experiment and 2 d after the end of the experiment, 5 mL of elbow venous blood was extracted from all participants. The supernatant was collected and stored at −80°C for further use.

### Psychological Evaluation

Psychological questionnaires were performed before and after the waterfall forest or urban programs. The Hamilton Anxiety Scale (HAMA) and Hamilton Depression Scale (HAMD) were used to evaluate anxiety and depression and their severity for all the participants. The Fatigue Scale-14 (FS-14) was used to evaluate fatigue severity.

### Flow Cytometry

Peripheral blood was collected for flow cytometry analysis. The COULTER Epics XL flow cytometer (Beckman Coulter, USA) was used to detect the number of T lymphocyte subsets (CD3, CD4, CD8) and NK cells (CD16). The expression of lymphocytes was analyzed by using software EXPO32 V1.2.

### Preparation of Chronic Stress Rat Model and Experimental Groups

All experimental procedures were approved by the Ethical Committee and the Animal Experimental Committee of Guizhou Provincial People's Hospital. All animals were handled in accordance with “The Guidance on the Care of Laboratory Animals.”

A total of 40 healthy male Wistar rats (weighing 180–200 grams) were purchased from the Animal Research Center of Central South University. All animals were housed in a temperature-controlled environment maintained at room temperature of 25 ± 2°C with a cycle of 12-h light and 12-h darkness and were given free access to food and water. All the rats were randomly divided into 4 equal groups: normal control group (NC, *n* = 10), chronic stress model group (CM, *n* = 10), waterfall forest environment group (WF, *n* = 10), and natural restoration group (NR, *n* = 10). The chronic stress rat model was established using a combination of high-fat and high-sugar diet plus forced cold water stimulation for 5 weeks. Rats in the NC group were given normal diets. Rats in the CM, WF, and NR groups were given high-fat diet (15% lard and 10% sucrose). After the successful establishment of the chronic stress rat model, rats in the WF group were acclimated to the water forest environment for 1 week and rats in the NR group were acclimated to the urban environment for 1 week. Sucrose water consumption in each group was detected before and after the establishment of the chronic stress rat model. At the end of the experiment, blood was taken from the aorta abdominalis of rats anesthetized by 10% chloral hydrate (Wuhan Boster Biological Technology, Ltd., Wuhan, China) and liver tissues were also collected. Samples were frozen in liquid nitrogen and stored at −80°C for further study.

### Assessment of Physiological Indexes and Antioxidative Capacity

The content of glucose (GLU), triglyceride (TG), cholesterol (CHO), and uric acid (UA) were detected by using the biochemical analyzer (Mindray, BS-400, Shenzhen, China). The expression levels of adrenocorticotropic hormone (ACTH), cortisol (CORT), 5-hydroxytryptamine (5-HT), glutathione peroxidase (GSH-Px), superoxide dismutase (SOD), and malondialdehyde (MDA) were detected by using the enzyme-linked immunosorbent assay (ELISA) (Jiangsu Zeyu Biotechnology Corporation Ltd, Jiangsu, China).

### Hematoxylin–Eosin (HE) Staining

All rats were euthanized 1 week after the establishment of chronic stress rat model, and the liver tissues were collected. The liver tissues were fixed in 10% formaldehyde solution, placed in the embedding box, embedded in paraffin, and then cut into pieces with 1.5 cm × 1.5 cm × 4 μm. The staining process was carried out as follows: hematoxylin staining, eosin staining, transparency, and sealing. Finally images were collected and analyzed.

### Immunohistochemical Staining

Liver tissues were put in the oven at 60°C for 24 h. Then, liver tissue slides were deparaffinized, dehydrated in alcohol, and washed with phosphate-buffered saline three times. Following this, antigen retrieval was conducted by boiling the sections in 0.01 M sodium citrate buffer. The sections were blocked with 5% BSA and incubated with a primary antibody (rabbit anti-rat NOX4 polyclonal antibody) (1:100; Abcam, MA, USA) overnight at 4°C followed by incubation with a secondary antibody (1:100; Abcam, MA, USA) for 1 h at room temperature. Finally, DAB and hematoxylin staining was performed. NOX4-positive areas were examined and observed using an Olympus BX53 fluorescence microscope (Tokyo, Japan).

### Immunofluorescence Staining

For immunofluorescence, the cultured cells were fixed with 4% paraformaldehyde and permeabilized with 1% Triton X-100 for 10 min. Then, cells were incubated with anti-NOX4 and anti-NF-κB p65 at 4°C overnight, followed by incubation of Alexa Fluor 594 conjugated secondary antibody at room temperature for 1 h. Confocal images were obtained using a Nikon C2 Plus confocal microscope (Tokyo, Japan).

### Measurement of ROS

To detect the production of ROS in the liver tissues, cells were incubated in 10 μM 2,7-dichlorofluorescein diacetate (DCFH-DA) (DCFH-DA, Sigma-Aldrich, USA) on a shaker at 37°C for 30 min. Then, cells were washed with PBS for 3 times and 4′,6-diamidino-2-phenylindole (DAPI; Thermo Fisher) was added to the cultures for nuclear staining. Fluorescence microscopy and fluorescence spectrophotometric analysis were used to determine the expression level of ROS.

### Quantitative Reverse Transcription PCR (qRT-PCR)

The liver tissues of rats were collected from all the groups. The total RNA was extracted using the TRIzol reagents (Invitrogen Life Technologies, Inc.). The microspectrophotometer (Tiangen Biotech Co., Ltd.) was used to detect the concentration and purity of RNA. Briefly, first-strand cDNA was synthesized using the RevertAid First Strand cDNA synthesis kit (Thermo Fisher Scientific, MA, USA). Then, qRT-PCR analyses of NOX4, NLK, TLR4, IKB, and NF-κB p65 mRNAs were performed with an ABI Q6 real-time PCR machine (Applied Biosystem Inc., MA, USA) using QuantiFast SYBR Green PCR Kit (Qiagen, Hilden, Germany) according to the instruction. Every sample was assayed in triplicates. Glyceraldehyde-3-phosphate dehydrogenase (GAPDH) was served as an internal control. The relative mRNA expression of NOX4, NLK, TLR4, IKB, and NF-κB p65 was determined by the 2-ΔΔCt methods. All primer sequences are displayed in Supplementary Table 1.

### Western Blot

The liver tissues were treated with RIPA lysis buffer (Thermo, USA) to extract total protein. The protein concentration was measured using a BSA kit (TaKaRa, Dalian). Then, ~20 μg protein was separated using 10% sodium dodecyl sulfate polyacrylamide gel electrophoresis and transferred onto a polyvinylidene fluoride (PVDF) membrane (Thermo Scientific, Madison, WI, USA). PVDF membranes were blocked with TBST solution containing 5% skim milk at room temperature for 3 h. The membranes were then incubated with primary anti-NOX4, anti-ub-NEMO, anti-ub-NLK, anti-TLR4, anti-p-IKB, anti-IKB, anti-NF-κB p65, and anti-IL-6 antibodies (1:100; Abcam, MA, USA) overnight at 4°C. Then, membranes were incubated with horseradish peroxidase-conjugated secondary antibody (1:5,000; Abcam, MA, USA) at room temperature for 1 h. Finally, images were scanned and analyzed using a Quantity One Imaging System.

### Statistical Analyses

Statistical analysis was performed using the SPSS 17.0 (Chicago, IL, USA). The data were expressed as mean ± standard deviation. One-way analysis of variance was used to determine the significant differences between different groups with an additional Turkey's *post hoc* test. Value of *p* < 0.05 was defined as statistically significant. Value of *p* < 0.01 was defined as extremely statistically significant.

A detailed description of the added methods can be found in Supplemental Materials.

## Results

### Participants' Characteristics

This study included 24 patients with chronic fatigue, including 15 women (mean BMI 22.1 ± 1.8) and 9 men (mean BMI 21.9 ± 2.1), with an average age of 42.3 years for women and 39.4 years for men. The demographic characteristics of the WF and U groups of participants are shown in Table 1. There was no statistical difference in basic characteristics and lifestyle between the two groups.

**Table 1:** Characteristics of patients with chronic fatigue at study start.

**Parameters**	**Urban group**	**Waterfall forest environment group**	***p***
Variables	*N* = 12 (male=5)	*N* = 12 (male=4)	
Age (years)	39.58 ± 7.95	42.58 ± 12.15	0.48
BMI (kg/m2)	22.578 ± 1.832	22.411 ± 2.054	0.84
Waist circumference (cm)	68.5 ± 9.73	71.54 ± 10.14	0.46
Systolic BP (mmHg)	114.75 ± 13.67	115.83 ± 16.94	0.86
Diastolic BP (mmHg)	78.83 ± 7.76	74.58 ± 8.60	0.22
Cholesterol (mmol/L)	5.07 ± 0.89	4.99 ± 0.62	0.78
Triglycerides (mmol/L)	2.24 ± 1.30	2.19 ± 1.43	0.92
Creatinine(μmol/L)	70.75 ± 13.08	70.90 ± 14.29	0.98
Average sleep time (h/day, last 3 months)	7.24 ± 0.67	7.06 ± 1.00	0.6
Coffee (%)	42	58	<0.001
≥360 ml/day	25	8.3	
<360 ml/day	17	50	
Tea (%)	41	41	
≥500 ml/day	25	8.3	<0.001
<500 ml/day	16.7	33.3	
Alcohol drinking (%)	16.7	25	<0.001
≥20 g/day	8.3	8.3	
<20 g/day	8.3	16.7	
Smoking (%)	33.3	16.7	
Ex-smoker	0	0	
Current	33.3	16.7	
Drug abuse (marijuana, cocaine, psychoactive)	0	0	
Exercise (%)	0.92	0.67	<0.001

*BMI, body mass index; BP, blood pressure*.

### The Waterfall Forest Environment Improves Psychological Symptoms and Physiological Indexes

**Figure 1:**
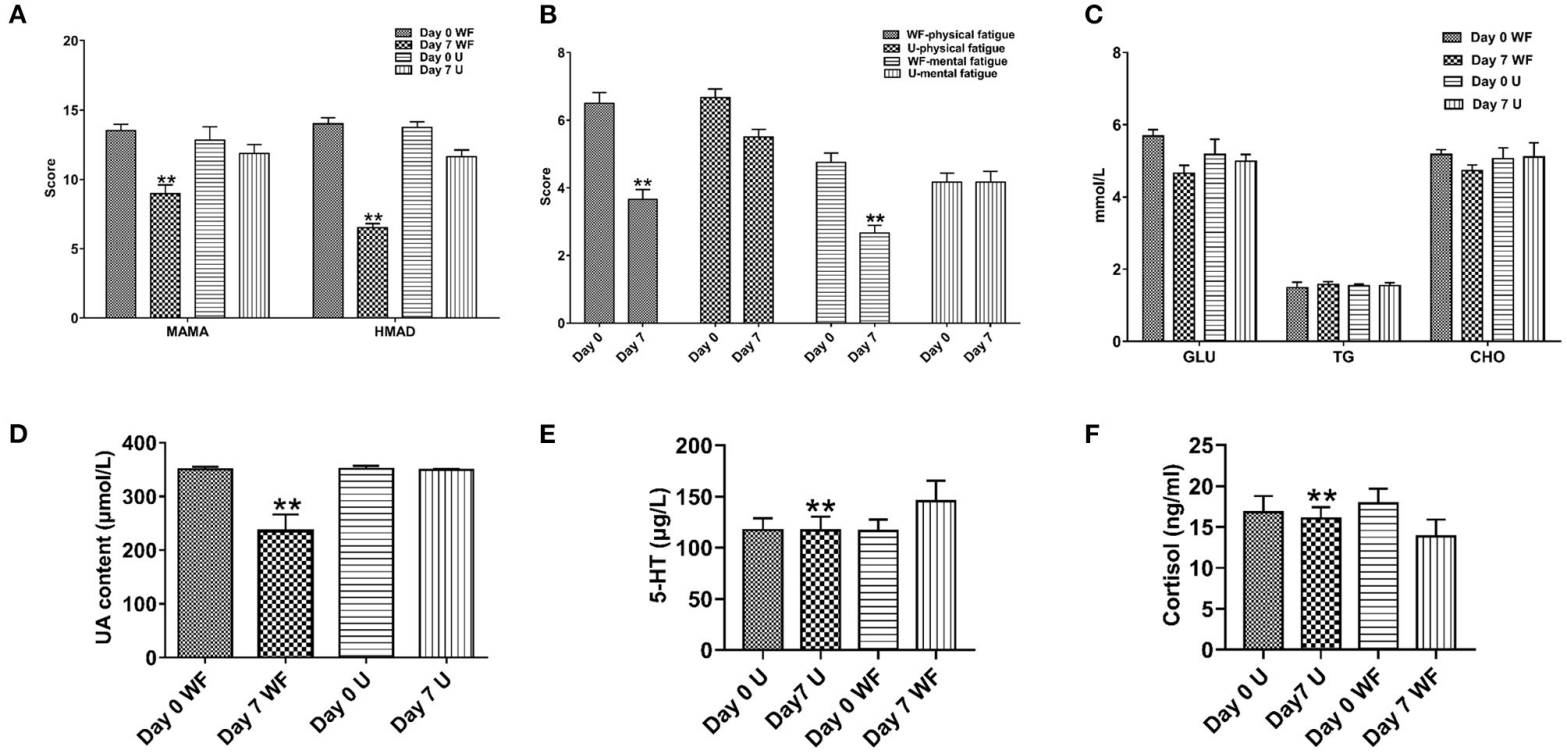
Effects of the waterfall forest environment (WF) on psychological symptoms and physiological indexes. **(A)** Hamilton Anxiety Scale (HAMA) and Hamilton Depression Scale (HAMD). **(B)** Fatigue Scale-14 (FS-14). **(C)** Effects of the WF on the content of serum glucose (GLU), triglycerides (TG), and total cholesterol (CHO). **(D)** Effects of the WF on the content of uric acid (UA). **(E)** Effects of the WF on the level of 5-hydroxytryptamine (5-HT). **(F)** Effects of the WF on the level of cortisol (CORT). U represents the urban group. **p < 0.01, Day 7 WF vs. Day 7 U.

To evaluate the effect of the WF on psychological symptoms of patients with chronic fatigue, HAMA, HMAD, and FS-14 were evaluated. As shown in Figure 1A, in comparison with 7 days of intervention of the urban environment, the HAMA and HMAD scores were significantly decreased after 7 days of intervention of the WF. In addition, FS-14 has 2 components including physical fatigue and mental fatigue. The WF group showed lower scores in physical fatigue and mental fatigue than the U group after 7 days (Figure 1B). These data indicated that the WF could improve psychological symptoms in patients with chronic fatigue. In addition, the levels of physiological indexes were determined using ELISA. The levels of GLU, TG, and CHO were decreased after 7 days of WF therapy compared with 7 days of intervention of the urban environment (Figure 1C). Meanwhile, the WF group showed a significant decrease in the level of UA compared with the U group after 7 days (Figure 1D). Moreover, we discovered that the 5-HT level was obviously higher in the WF group than in the U group after 7 days of intervention. However, the WF group showed a significant decrease in the expression of CORT compared with the U group after 7 days of intervention (Figures 1E,F).

### The Waterfall Forest Environment Improves Cognitive Function

To evaluate the effect of the WF on cognitive function of patients with chronic fatigue, the Stroop test and PASAT test were performed. As shown in Supplementary Table 2, patients with chronic fatigue in the WF group achieved higher scores in the word test of the Stroop test than those in the U group after 7 days of intervention. There was no significant difference in the color test and color word interference test between the WF group and the U group. Meanwhile, patients with chronic fatigue in the WF group answered more correct questions than those in the U group after 7 days of intervention.

### The Waterfall Forest Environment Improves Antioxidant Ability and Immunity

**Figure 2:**
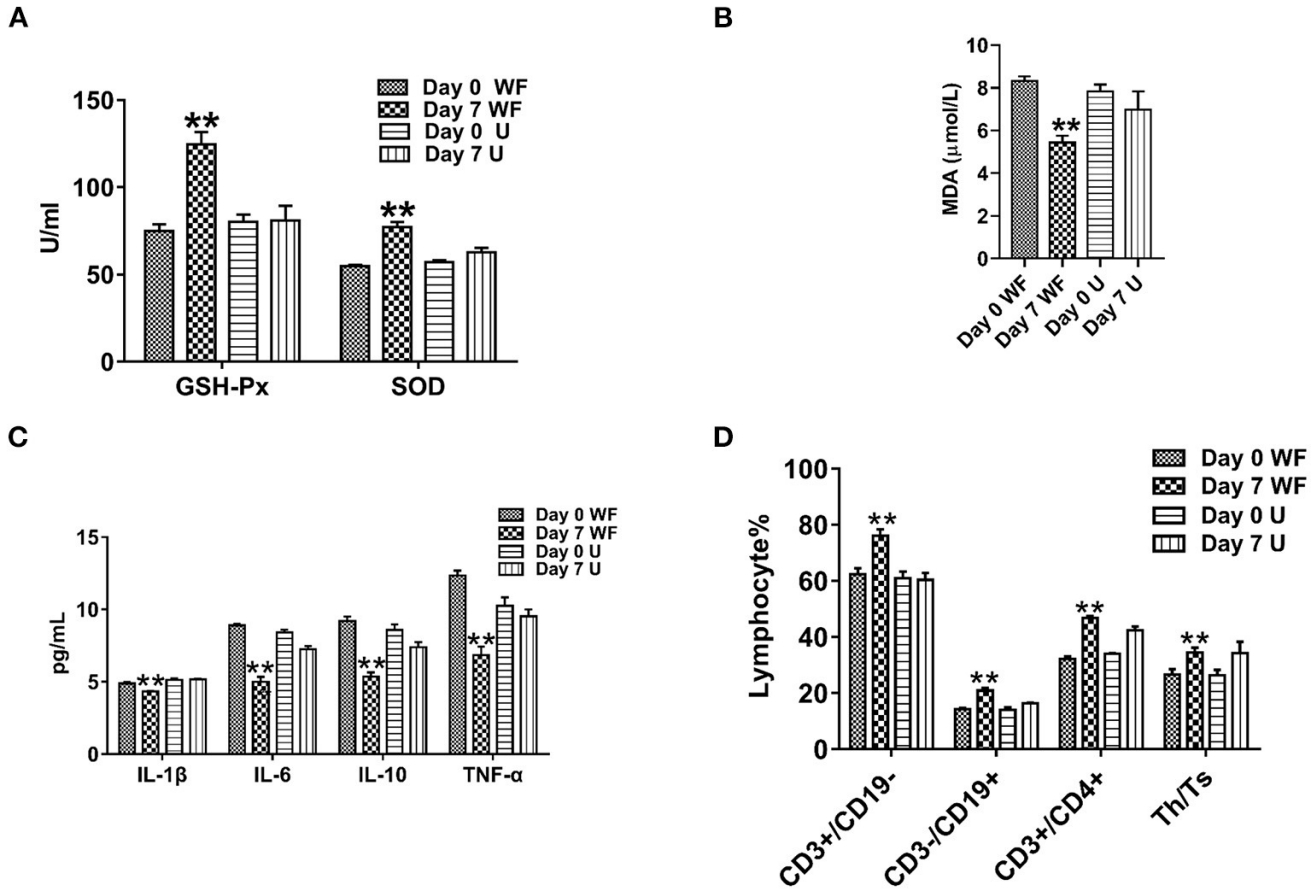
Effects of the waterfall forest environment (WF) on antioxidant indexes and immunity. **(A)** Glutathione peroxidase (GSH-Px) and superoxide dismutase (SOD) activity. **(B)** Malondialdehyde (MDA) content. **(C)** Effects of the WF on the expression of inflammatory factors. **(D)** Effects of the WF on immune levels. **p < 0.01, Day 7 WF vs. Day 7 U.

To study the effect of the WF on the antioxidant capacity of patients with chronic fatigue, GSH-Px, SOD, and MDA activities were detected. The levels of GSH-Px and SOD in serum of participants were remarkably enhanced in the WF group after 7 days of intervention compared with the U group (Figure 2A). However, the level of MDA was obviously lower after 7 days of intervention of the WF group compared with the U group (Figure 2B). In addition, the levels of inflammatory factors in all the groups were detected. The WF group showed a significant decrease in the expression of IL-1β, TNF-α, IL-6, and IL-10 compared with the U group after 7 days of intervention (Figure 2C). In addition, changes of T lymphocyte subsets and NK cell levels before and after environmental intervention were detected using flow cytometry. After treatment in the WF group for 7 days, the number of B lymphocytes, T cells, NK cells, and Th/Ts ratio in peripheral blood was dramatically increased compared with the U group (Figure 2D).

### The Waterfall Forest Environment Improves Hopelessness of Chronic Stress Rats

**Figure 3:**
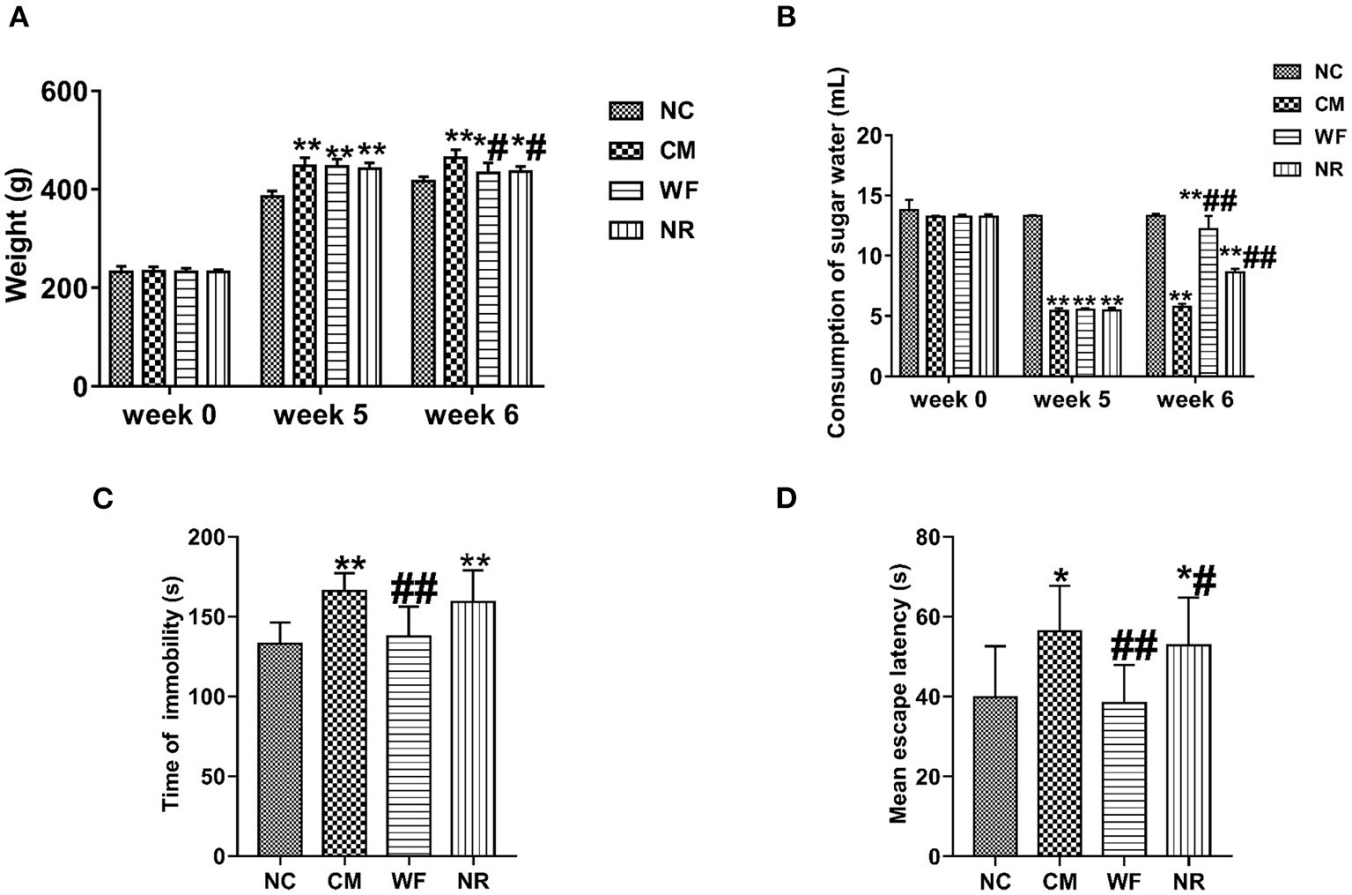
The waterfall forest environment (WF) improves hopelessness of chronic stress rats. **(A)** Effects of WF on body weight of rats in all the groups. **(B)** Effects of WF on the consumption of sugar water in all the groups. **(C)** Effects of WF on immobility time of rats in the tail suspension test. **(D)** Effects of WF on escape latency of rats in the Morris water maze test. *p < 0.05 and **p < 0.01 vs. the normal control (NC) group. ^#^*p* < 0.05 and ^##^*p* < 0.01 vs. the chronic stress model (CM) group.

In order to further determine the effect of the WF in the improvement of chronic stress, the chronic stress rat model was established. After 5 weeks of chronic stress modeling, the weight of the rats in the CM group was significantly increased compared with that in the NC group (Figure 3A). The sugar water consumption test indicated that the sugar water consumption in the CM group was significantly lower than that in the NC group, which proved that the rat model was successfully constructed. After 7 days of treatment in the WF, sugar water consumption returned to the normal level (Figure 3B). In addition, we performed behavioral tests to verify the behavioral changes of different groups. The result of the TST test revealed that rats in the CM group showed a remarkable increased immobility time compared with the NC group (Figure 3C). The result of the MWM test indicated that rats in the CM group took a longer time to find the platform compared with the NC group (Figure 3D). However, After 7 days of treatment in the WF, immobility time in the TST test and time to find the platform in the MWM test of chronic stress rats were dramatically reduced. The results indicated that the WF could improve the hopelessness of chronic stress rats.

### The Waterfall Forest Environment Reduces the Damage of Chronic Stress to Liver and Excitability of Neurons

The liver tissues of rats from all the groups were observed under the light microscope through HE staining. As shown in Supplementary Figure 1A, in the NC group, the liver lobules were intact, the hepatocyte cords were arranged neatly, and the liver cells had no lesions. In the CM group, the liver cells showed edema, balloonning degeneration, and partial steatosis. A small amount of inflammatory response in the portal area was observed. In the WF group, the denatured liver cells gradually decreased, the inflammation in the portal area improved, and the structure of the liver cells gradually returned to normal. In addition, rats in the CM group exhibited a significant increase in the expression levels of ACTH and CORT compared with the NC group, while the WF intervention reversed these alterations (Supplementary Figures 1B,C), indicating that the WF could reduce excitability of neurons of chronic stress rats.

### The Waterfall Forest Environment Reduces ROS Accumulation

**Figure 4:**
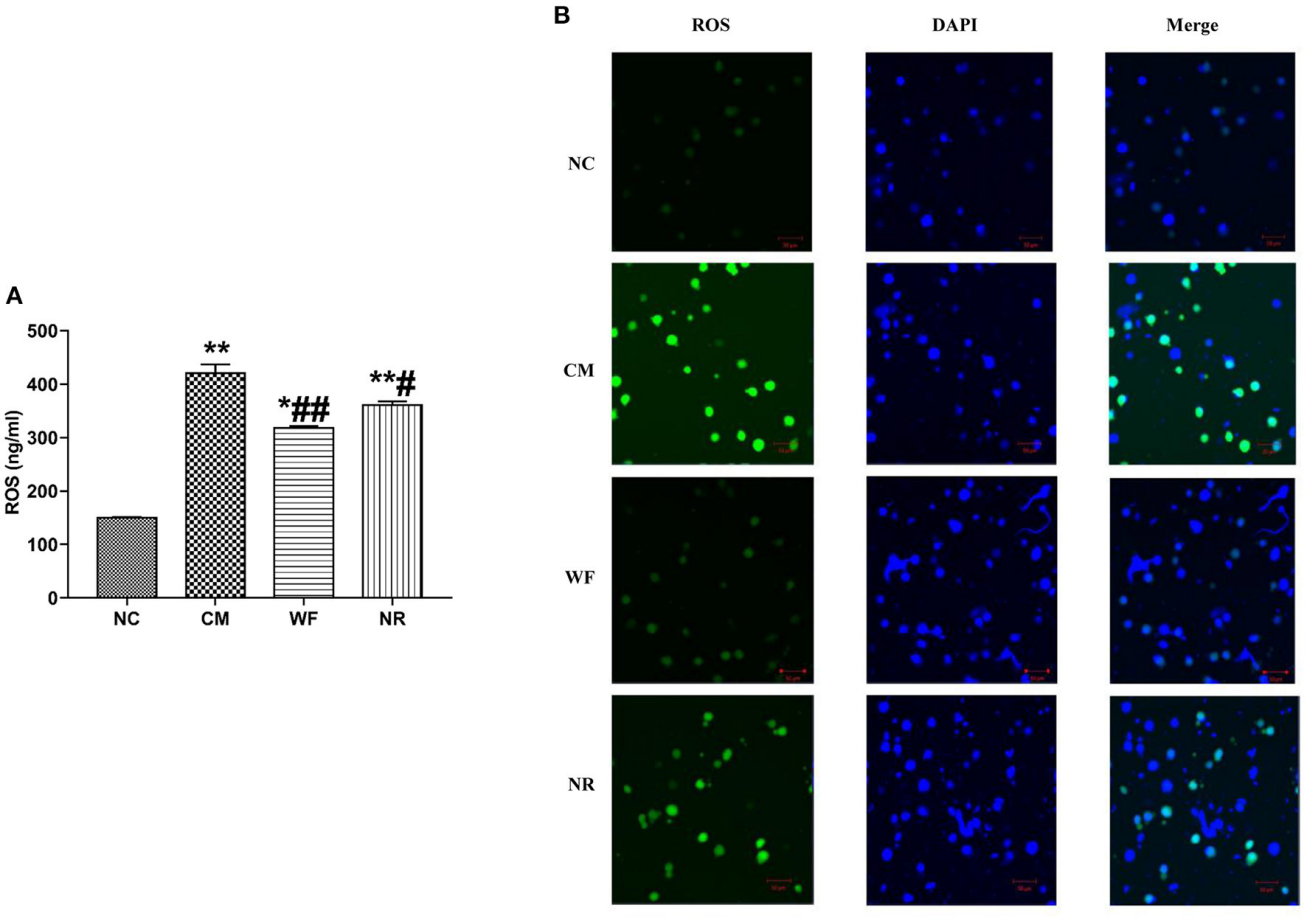
Effects of the waterfall forest environment (WF) on the expression of reactive oxygen species (ROS). **(A)** Effects of the WF on the expression of ROS in serum of rats. *p < 0.05 and **p < 0.01 vs. the normal control (NC) group. ^#^*p* < 0.05 and ^##^*p* < 0.01 vs. the chronic stress model (CM) group. **(B)** Effects of the WF on the expression of ROS in liver tissues of rats. The production of ROS in the liver tissues was detected by using dichlorofluorescein diacetate (DCFH-DA). Scale bars are 50 μm.

To investigate the effect of the WF on ROS accumulation, the content of ROS in the serum and liver tissues was detected. The content of ROS in the serum in the CM, NR, and WF groups was remarkably increased compared with that in the NC group. However, in comparison with the CM group, the content of ROS in the serum in the WF and NR group was significantly reduced. At the same time, the content of ROS in the serum in the WF group was significantly lower than that in the NR group (Figure 4A). It indicated that the therapeutic effect of WF was better than that of NR. In addition, DCFH-DA was used to detect the content of ROS in the liver tissues. ROS content in the CM group was higher than that in the NC group. The WF group had less ROS content compared with the NR group (Figure 4B). It revealed that the WF could reduce ROS accumulation in liver tissues, thus reducing the degree of damage caused by free radicals to liver tissues.

### The Waterfall Forest Environment Inhibits the Expression of NOX4

**Figure 5:**
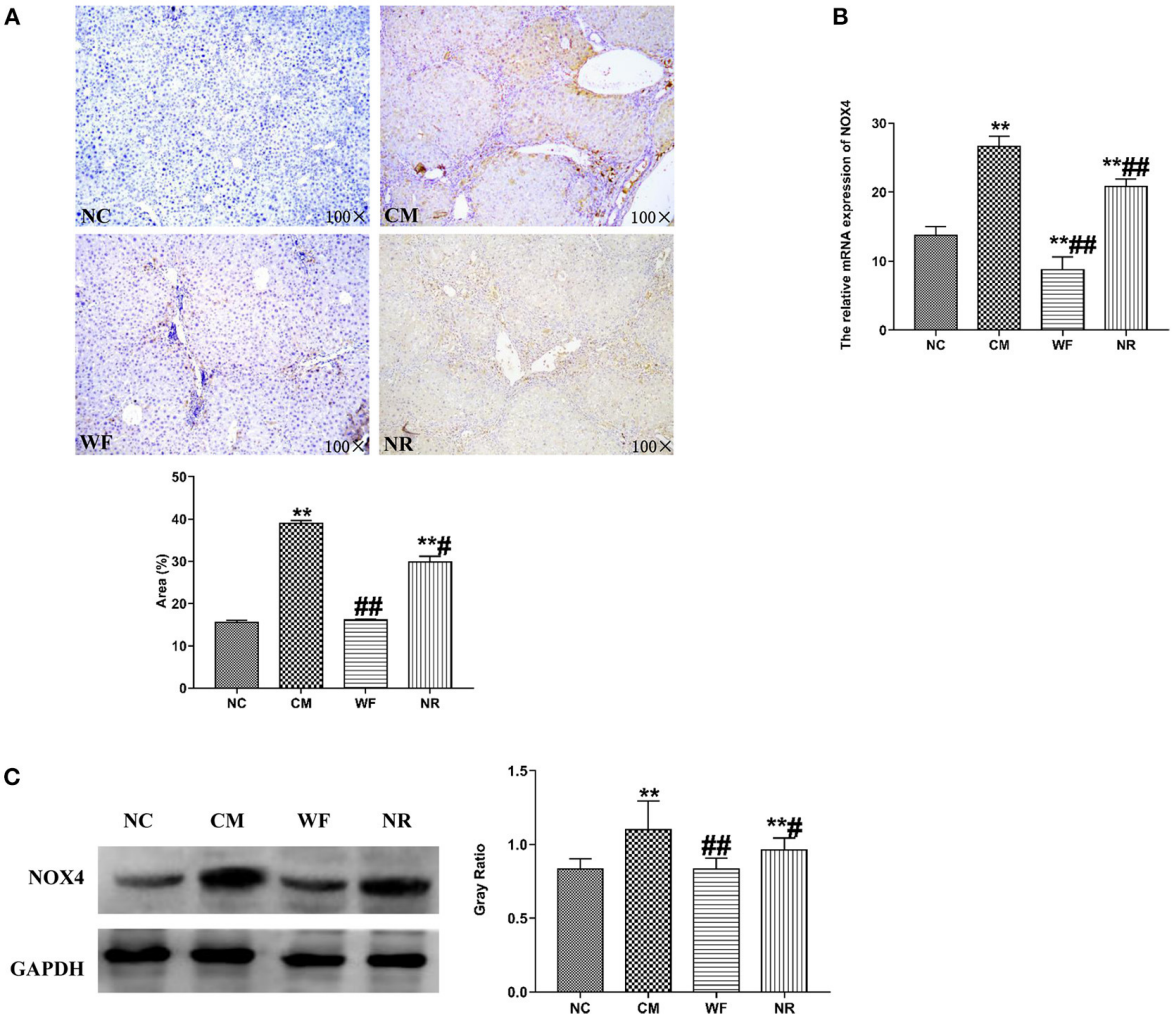
The waterfall forest environment (WF) reduced liver damage by inhibiting the level of NOX4. **(A)** The expression of NOX4 was detected by immunohistochemical staining. Scale bars are 100 ×. **(B)** Effects of the WF on the mRNA expression of NOX4. The relative mRNA expression of NOX4 in the liver tissues was detected by qRT-PCR. **(C)** Effects of the WF on the protein expression of NOX4. The relative protein expression of NOX4 in the liver tissues was detected by western blot. GAPDH was used as an internal control. **p < 0.01 vs. the normal control (NC) group. ^#^p < 0.05 and ^##^*p* < 0.01 vs. the chronic stress model (CM) group.

**Figure 6:**
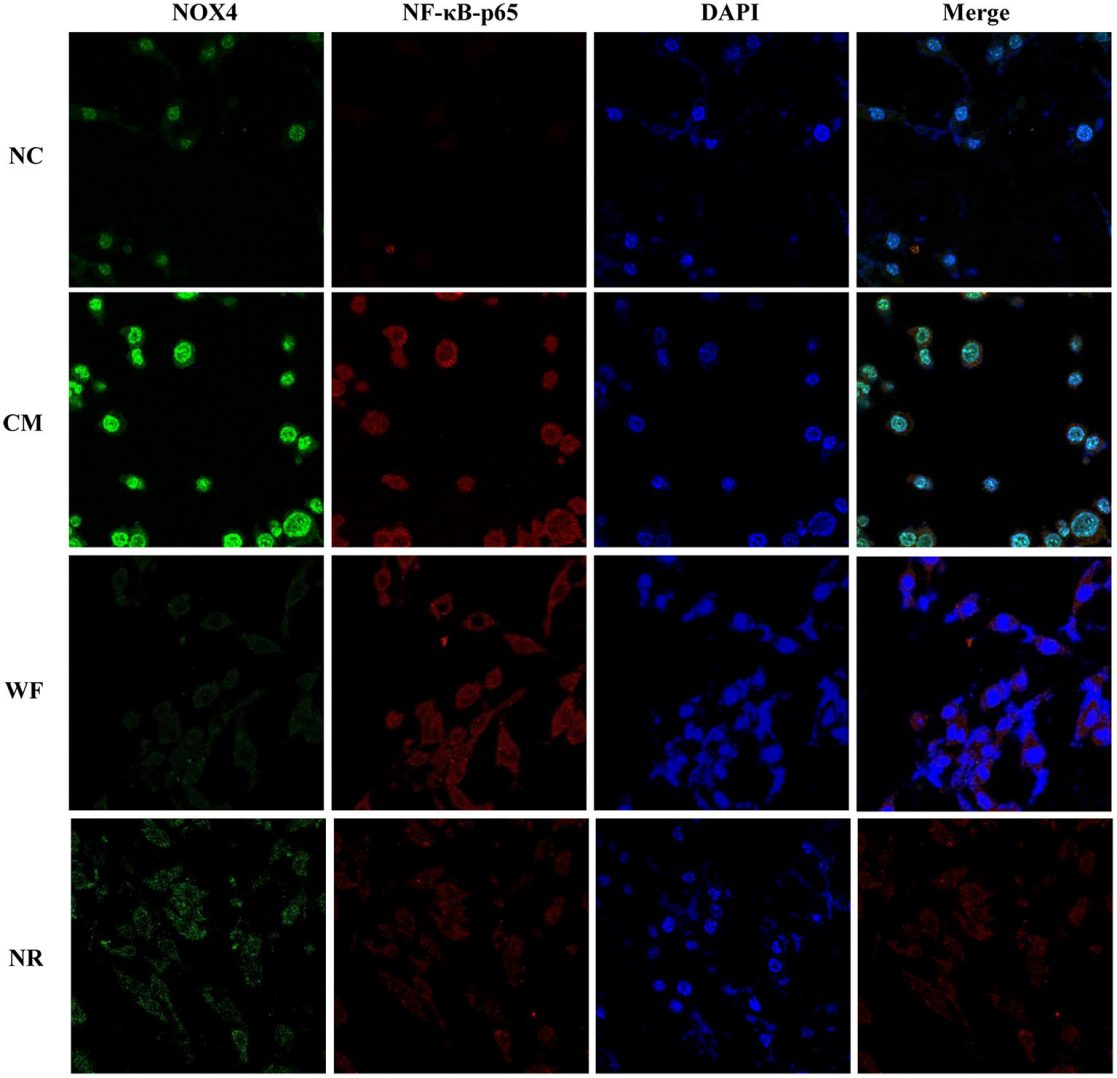
Effects of the waterfall forest environment (WF) on the expression of NOX4 and NF-κB p65. The expression of NOX4 and NF-κB p65 was detected by immunofluorescence staining. Scale bars are 50 μm.

The expression of NOX4 in liver tissues of all the groups was detected. The immunohistochemical staining showed that there was almost no yellow positive expression in the NC group (Figure 5A). The CM group showed a strongly positive expression of NOX4, which was mostly distributed in the portal area and around the central vein. In the NR group, the expression of NOX4 in liver tissues was reduced compared with the CM group. In the WF group, a small number of fine and discontinuous yellow fibers were observed in liver tissues and the expression of NOX4 was weakly positive, which was similar to that in the NC group, indicating that the WF could reduce liver damage induced by chronic stress. In addition, the immunofluorescence test showed that there was almost no fluorescent expression of NOX4 in the NC group (Figure 6). In comparison with the NC group, the fluorescent expression of NOX4 was significantly increased in the CM group. However, the WF group showed a significant decrease in the fluorescent expression of NOX4 compared with the CM group. Moreover, qRT-PCR results showed that the mRNA expression levels of NOX4 in the CM group were dramatically higher than those in the NC group. However, the WF group reversed these changes. In comparison with the CM group, the mRNA expression levels of NOX4 in the NR group was prominently decreased (Figure 5B), but the effect of the NR was lower than that of the WF group. Furthermore, the CM group showed a significant increase in the protein expression level of NOX4 compared with the NC group (Figure 5C). However, the WF group reversed these changes. The results indicated that the WF reduced liver damage by inhibiting the expression of NOX4.

### The Waterfall Forest Environment Reduces Liver Damage by Inhibiting the NF-κB Signaling Pathway

**Figure 7:**
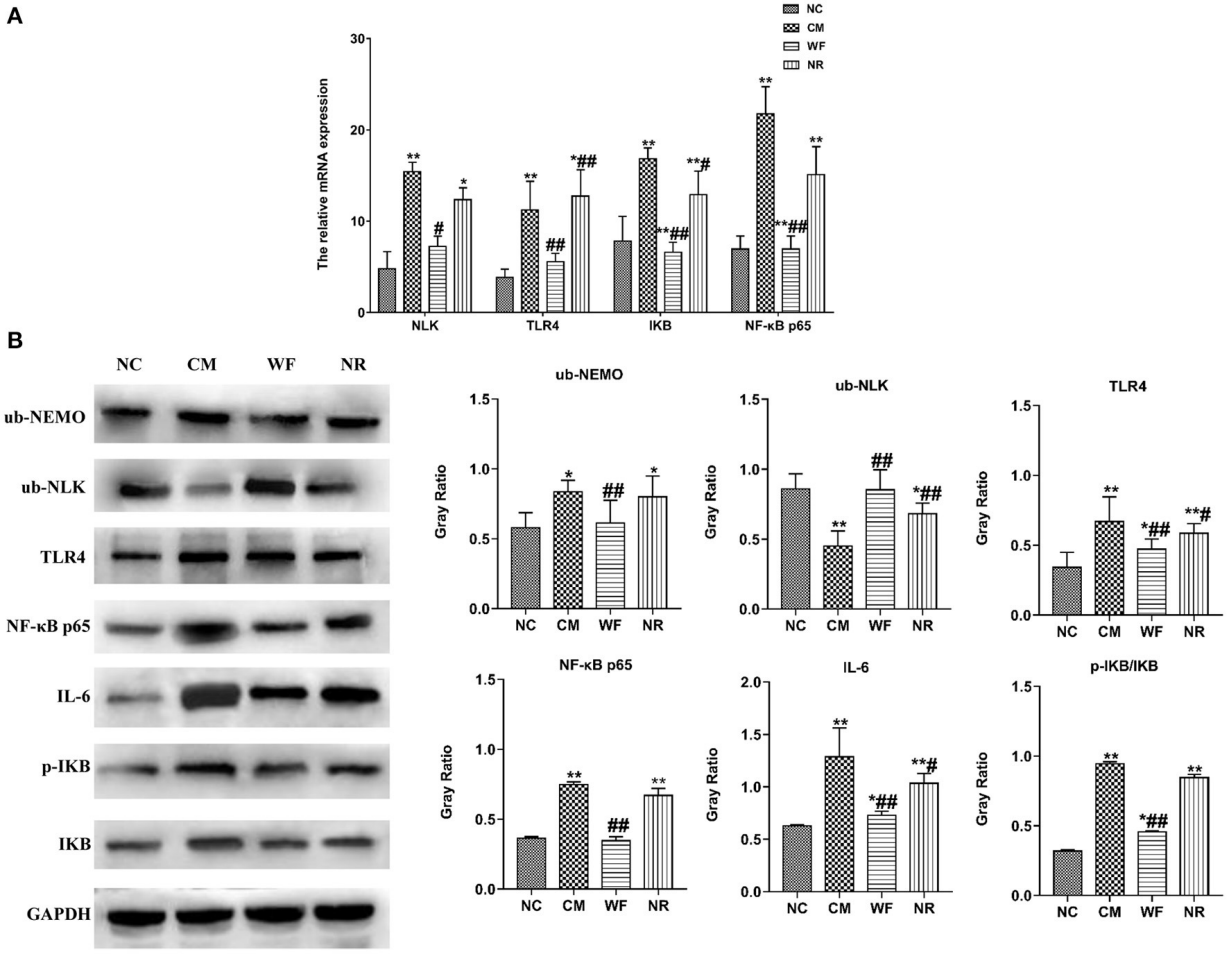
The waterfall forest environment (WF) reduced liver damage by inhibiting the NF-κB signaling pathway. **(A)** Effects of the WF on the mRNA expression of NLK, TLR4, IKB, and NF-κB p65. The relative mRNA expression of NLK, TLR4, IKB, and NF-κB p65 in the liver tissues was detected by qRT-PCR. **(B)** Effects of the WF on the protein expression of NF-κB p65, IL-6, p-IKB, and IKB. The relative protein expression of ub-NEMO, ub-NLK, TLR4, NF-κB p65, IL-6, p-IKB, and IKB in the liver tissues was detected by western blot. *p < 0.05 and **p < 0.01 vs. the normal control (NC) group. ^#^*p* < 0.05 and ^##^*p* < 0.01 vs. the chronic stress model (CM) group.

To understand the mechanism by which the WF alleviates chronic stress, the expression of indicators related to the NF-κB pathway was detected. The immunofluorescence test showed that there was little fluorescent expression of NF-κB p65 in the NC group (Figure 6). In comparison with the NC group, the fluorescent expression of NF-κB p65 was significantly enhanced in the CM group. However, the WF group showed a remarkable decrease in the fluorescent expression of NF-κB p65 compared with the CM group, indicating that the phosphorylation of NF-κB p65 was reduced, thus inhibiting inflammation. In addition, qRT-PCR results showed that the mRNA expression levels of NLK, TLR4, IKB, and NF-κB p65 in the CM group were significantly higher than those in the NC group. However, the WF group reversed these changes. Compared with the CM group, the mRNA expression levels of these indicators in the NR group were prominently decreased (Figure 7A), while the effect of the NR was lower than that of the WF. Moreover, the CM group showed a significant increase in the protein expression levels of ub-NEMO, TLR4, NF-κB p65, IL-6, p-IKB, and IKB compared with the NC group (Figure 7B). However, the WF group reversed these changes. In contrast, the protein expression level of ub-NLK was obviously reduced in the CM group compared with the NC group, while the WF group reversed the alteration. These results indicated that WF might reduce liver damage by inhibiting the NF-κB signaling pathway.

## Discussion

With the acceleration of modern urbanization, the prevalence of chronic fatigue and physiological and psychological diseases in human beings is gradually increasing (22). Forest therapy is recognized as a fast-growing therapeutic method for improving depression and anxiety symptoms of urban people (23). A growing number of researches have provided evidence for the validity of forest therapy in a variety of physiological and psychological symptoms. Forest therapy has been reported to be effective in reducing stress, anxiety, and depression symptoms (24). It has also been shown to lower sympathetic nervous system activity, pulse rate, and blood pressure (25). Similarly, in the current study, the WF significantly improved psychological symptoms of patients with chronic fatigue compared with the urban environment, as demonstrated by the decreased levels of HAMA, HMAD, and FS-14 scores. Compared with the forest therapy, the WF has more advantages in treating chronic fatigue. Forest and waterfall both can have positive effects on psychological symptoms. The waterfall has been proven to improve depression, anxiety, interpersonal sensitivity, and paranoid ideation symptoms in people with moderate to high stress levels (15). In addition, the Stroop test and PASAT test were found to evaluate cognitive function (26). We discovered that the WF resulted in a significant increase in the scores achieved in the word test of the Stroop test, and the PASAT test. Collectively, the results indicated that the WF can effectively treat chronic fatigue.

Chronic fatigue is a health state of low quality between health and disease. Clinically, it is difficult to find substantive lesions in patients with chronic fatigue, but there may be potential changes in physiological or metabolic indicators (27, 28). In this study, it was found that the blood uric acid level of the participants was significantly lower after treatment in the waterfall forest environment than that before the treatment. Studies have shown that chronic stress could increase the body's free fatty acids and further lead to the production of uric acid. The meta-analysis also indicated that there was a correlation between uric acid metabolism and stress (29–31). Therefore, it was speculated that the WF had a positive effect on reducing body pressure level. In addition, we found that the WF remarkably increased the levels of GSH-PX and SOD in serum of participants compared with the urban environment after 7 days of intervention. GSH-PX and SOD are important antioxidant enzymes, which can help remove superoxide free radicals to avoid oxidative damage (32). Studies have reported that the levels of endogenous antioxidant enzymes were enhanced in people exposed to the forest environment (33). Therefore, the results indicated that the WF could enhance the antioxidant ability of patients with chronic fatigue.

Chronic fatigue is associated with the production of inflammatory cytokines such as IL-6, IL-10, IL-13, and TNF-α (34). A recent study has shown that the levels of TNFα, IL-2, and IL-4 were remarkably increased in patients with CFS compared with the control group (35). Another study reported that there was a positive correlation between depression severity in patients with CFS and IL-6 levels (36). Moreover, Hardcastle et al. reported that patients with severe CFS had elevated IL-6 levels and patients with moderate CFS had relatively lower IL-6 levels (37). Furthermore, it was found that the levels of IL-10 were higher in patients with CFS than those in the healthy control group (38). In our study, patients with chronic fatigue had significant decreased IL-1β, TNF-α, IL-6, and IL-10 levels after 7 days of exposure to the WF compared with the urban environment. It indicated that the WF could improve inflammatory response of patients with chronic fatigue. At the same time, T regulatory (Treg) cells and NK cells have been shown to play a role in the development of chronic fatigue (39, 40). The CD4+/CD8+ (Th/Ts) ratio can indicate the stability of the immune system of the body. When the ratio increases, the immune function of the body is enhanced (41). The number of NK cells is also an important indicator of immune function (42). In the present study, patients with chronic fatigue intervened by the WF showed the higher number of T cells, NK cells, and Th/Ts ratio in peripheral blood, which was dramatically higher than before the intervention. These results suggested that the WF can improve immunity of patients with chronic fatigue.

The mechanism of chronic fatigue has focused on the feedback regulation of the hypothalamic-pituitary-adrenal (HPA) axis (43, 44). During the neuroendocrine response, stress activates the HPA axis, leading to the disorder of metabolism of nutrients and the imbalance of homeostasis of the internal environment. The hypothalamus is the regulatory center of the HPA axis, and it is closely related to hormone secretion. ACTH and CORT are important indicators reflecting the stability and balance of the HPA axis. Stress first gathers in hypothalamic neurons, causing the excitability of the HPA axis to increase, and releasing ACTH and CORT. ACTH can stimulate the adrenal cortex to excessively release glucocorticoids, and large amounts of glucocorticoids can cause certain harm to the body (17). CORT plays an obvious role in regulating human metabolic function, correcting cognitive impairment, and regulating emotions such as fear and anxiety (45). In this study, CORT content of patients with chronic fatigue in the WF group and ACTH and CORT content of rats in the WF group were both lower than those in the U group. Therefore, it was speculated that the WF might improve chronic fatigue via maintaining the functional stability of the HPA axis.

In the present study, we also found that the expression levels of NOX4 and ROS in chronic stress rats were significantly downregulated after intervention of the WF. NOX4 is a kind of enzymes that mainly produces ROS in cells, and ROS is related to liver damage (20, 46). ROS can directly destroy the mitochondrial membrane, resulting in increased permeability of mitochondrial membrane, presenting with edema, showing DNA fragmentation, and finally resulting in cell necrosis (20, 47). Therefore, the results indicated that the WF may reduce the excessive ROS oxidation by reducing the expression of NOX4, thus achieving the purpose of liver protection. In addition, the NF-κB signaling pathway is a downstream pathway of ROS and regulates inflammation and apoptosis (48). The NF-κB pathway is activated when the body is attacked by bacteria, viruses, and inflammation (49). Activation of NF-κB is mainly dependent on IKB phosphorylation. NF-κB is a dimer (p65/p50) in a static state (50). Studies have shown that activated IKB kinase phosphorylates its substrate IKB, and p65 is also phosphorylated, further resulting in the nuclear transcription and regulation of inflammatory cytokine expression (51). In this study, the expression levels of IKB and NF-κB p65 in the WF group were significantly decreased compared with the CM group. It indicated that the WF could reduce IKB phosphorylation and NF-κB p65 expression, thus inhibiting the NF-κB signaling pathway and reducing the expression of downstream inflammatory factors.

In this study, there are several limitations. First, we have a small sample size. We have just investigated 24 patients with chronic fatigue and evaluated the effect of the WF on psychological symptoms of patients with chronic fatigue. Further study is needed to investigate more patients with chronic fatigue for obtaining more reliable outcomes. Second, participants in the present study were of different sexes, while this factor was not analyzed. It will be studied in future plans. Third, we did not provide appropriate control groups (without control in which participants only lived in the hotel near the core scenic area of Huangguoshu Waterfall and did not perform daily activity in the WF). Thus, although we observed pre- and post-effects, whether it was due to true effect is remains unknown. Therefore, it is necessary to consider these limitations in further studies.

## Conclusion

In summary, the WF improved psychological state and enhanced immunity in patients with chronic fatigue. In addition, it inhibited liver oxidative stress and damage in chronic sress rats by regulating the NOX4/ROS/NF-κB signaling pathway (Supplementary Figure 2). Our results showed that the WF had the potential to treat chronic fatigue.

## Data Availability Statement

The raw data supporting the conclusions of this article will be made available by the authors, without undue reservation.

## Ethics Statement

The studies involving human participants were reviewed and approved by the guidelines laid down in the Declaration of Helsinki and all procedures involving research study participants were approved by the Ethics Committee of Guizhou Provincial People's Hospital. Written informed consent was obtained from all subjects. Verbal consent was witnessed and formally recorded. The patients/participants provided their written informed consent to participate in this study. The animal study was reviewed and approved by the Ethical Committee and the Animal Experimental Committee of Guizhou Provincial People's Hospital. All animals were handled in accordance with The Guidance on the Care of Laboratory Animals.

## Author Contributions

ZZ, MC, and XL: conceptualization and funding acquisition. XZ and QO: data curation. ZZ, YW, YX, and SC: formal analysis. ZZ, XZ, YW, YX, and MZ: experimental studies. All authors are writing, original draft, review, and editing. All authors have read and agreed to the published version of the manuscript.

## Conflict of Interest

The authors declare that the research was conducted in the absence of any commercial or financial relationships that could be construed as a potential conflict of interest.

## Supplementary Material

Supplementary Table 1 Sequences of forward and reverse primers used in the qRT-PCR experiment.

Supplementary Table 2 Effects of the waterfall forest environment on cognitive function of patients with chronic fatigue. ^*^*p* < 0.05.

Supplementary Figure 1 The waterfall forest environment (WF) reduces the damage of chronic stress to liver, and excitability of neurons. **(A)** HE staining of the liver tissues in all the groups (100×). NC represents the normal control group. CM represents the chronic stress model group. WF represents the waterfall forest environment group. NR represents the natural restoration group. **(B)** Effects of WF on the level of adrenocorticotropic hormone (ACTH) in all the groups. **(C)** Effects of WF on the level of cortisol (CORT) in all the groups. ^*^*p* < 0.05 and ^**^*p* < 0.01 vs. the normal control (NC) group. ^#^*p* < 0.05 and ^##^*p* < 0.01 vs. the chronic stress model (CM) group.

Supplementary Figure 2 The waterfall forest environment inhibited chronic stress by regulating the NOX4/ROS/NF-κB signaling pathway.

## Abbreviations

WF: waterfall forest environment
U: urban
HAMA: Hamilton Anxiety Scale
HAMD: Hamilton Depression Scale
FS-14: Fatigue Scale-14
CFS: chronic fatigue syndrome
ROS: reactive oxygen species
NOX: nicotinamide adenine dinucleotide phosphate oxidase
5-HT: 5-hydroxytryptamine
ACTH: adrenocorticotropic hormone
CORT: cortisol
GLU: glucose
TG: triglyceride
CHO: cholesterol
UA: uric acid
GSH-Px: glutathione peroxidase
SOD: superoxide dismutase
MDA: malondialdehyde
ELISA: enzyme-linked immunosorbent assay
TST: tail suspension test
MWM: Morris water maze test
GAPDH: glyceraldehyde-3-phosphate dehydrogenase
NLK: NF-κB essential modulator (NEMO)-like kinase
TLR4: toll-like receptor-4
ub-NEMO: ubiquitinated NF-kappa-B essential modulator
ub-NLK: ubiquitinated NLK
ub-TLR4: ubiquitinated TLR4
NC: normal control
CM: chronic stress model
NR: natural restoration
DCFH-DA: dichlorofluorescein diacetate
HE: hematoxylin–eosin
qRT-PCR: quantitative reverse transcription PCR.
