# Supplementary material for: Waterfall Forest Environment Regulates Chronic Stress via the NOX4/ROS/NF-κB Signaling Pathway
Source: Front Neurol. 2021 Mar 18;12:619728. doi: 10.3389/fneur.2021.619728 (PMC8044934; doi:10.3389/fneur.2021.619728)
Supplement: Supplementary Table 1 — Sequences of forward and reverse primers used in the qRT-PCR experiment. [file Table_1.docx]

| **Gene name** | **Primer name** | **Primer sequence** |
| --- | --- | --- |
| GAPDH | GAPDH-F | 5’-GACGGCGACAGAGTCTCAT-3’ |
|  | GAPDH-R | 5’-GAACAGCATTCCCATCCCT-3’ |
| NOX4 | NOX4-F | 5’-CGGGTGGCTTGTTTGAAGTAT-3’ |
|  | NOX4-R | 5’-AAAACCCTCCAGGCAAAGAT-3’ |
| IKB | IKB-F | 5’-GAAACACTGGAAGCACGGAT-3’ |
|  | IKB-R | 5’-CAAGGTCAGAATGCACCAGA-3’ |
| NF-κB p65 | NF-κB p65-F | 5’-CCCAGCATCTCCACTCCGTC-3’ |
|  | NF-κB p65-R | 5’-ACATCAGCACCCAAAGTCACC-3’ |
| TLR4 | TLR4-F  TLR4-R | 5’-AGATCTGAGCTTCAACCCCCT-3’  5’-TGTCTCAATTTCACACCTGGAT-3’ |
| NLK | NLK-F  NLK-R | 5’-GAGTGAGATGGTGCAACCCA-3’  5’-CATCTGATTGCTCTGCCGGA-3’ |

**Table S1. Sequences of forward and reverse primers used in the qRT-PCR experiment.**
