# Supplementary material for: Waterfall Forest Environment Regulates Chronic Stress via the NOX4/ROS/NF-κB Signaling Pathway
Source: Front Neurol. 2021 Mar 18;12:619728. doi: 10.3389/fneur.2021.619728 (PMC8044934; doi:10.3389/fneur.2021.619728)
Supplement: Supplementary Table 2 — Effects of the waterfall forest environment on cognitive function of patients with chronic fatigue. *p < 0.05. [file Table_2.doc]

| **Index** | **Waterfall forest environment group** | **Urban group** | **p value** |
| --- | --- | --- | --- |
| Stroop test  Word test  Color test  Color word interference test  PASAT test  Correct number  Attempts number | 70.25±9.33  46.33±10.82  31.17±9.42  35.67±5.85  37.33±8.45 | 57.25±17.92*  39.08±9.67  24.33±8.66  28.17±7.30*  35.08±5.89 | 0.036*  0.07  0.15  0.01*  0.45 |

**Table S2 Effects of the waterfall forest environment on cognitive function of patients with chronic fatigue.**

*p<0.05.
