# Supplementary figures and images for: Waterfall Forest Environment Regulates Chronic Stress via the NOX4/ROS/NF-κB Signaling Pathway
Source: Front Neurol. 2021 Mar 18;12:619728. doi: 10.3389/fneur.2021.619728 (PMC8044934; doi:10.3389/fneur.2021.619728)

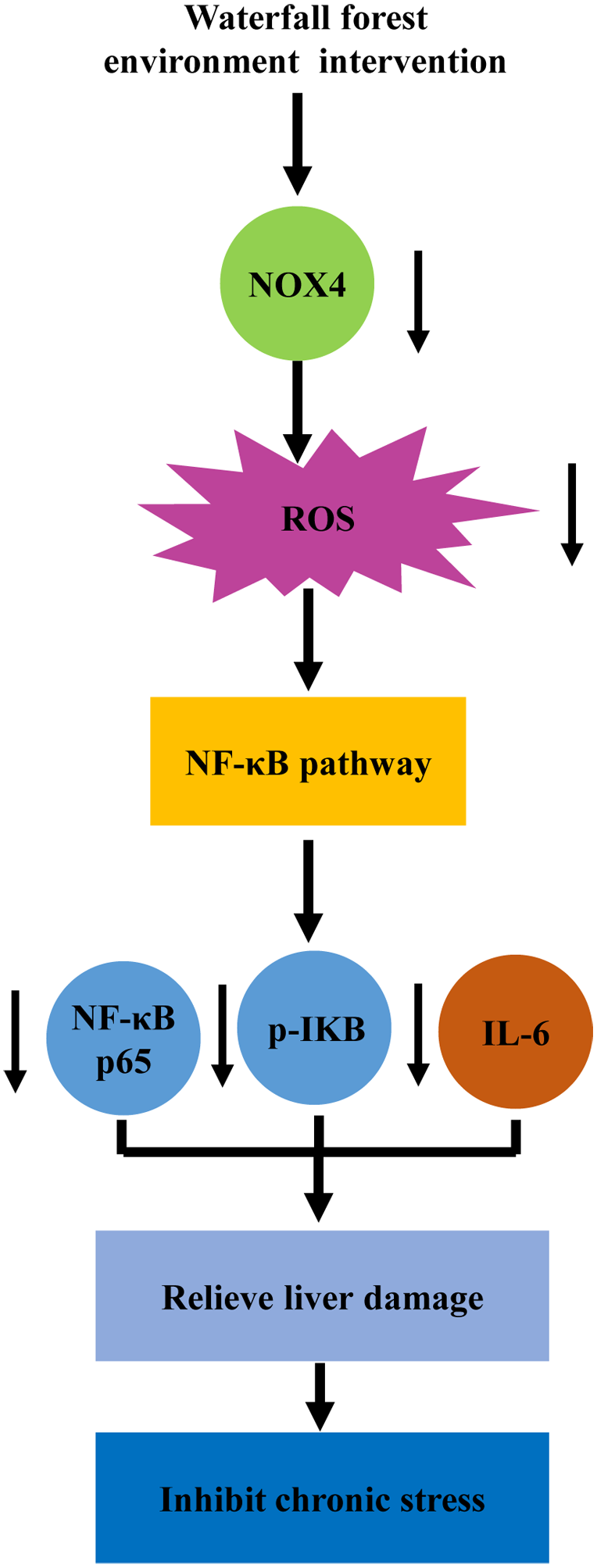

Supplement: Supplementary Figure 1 — The waterfall forest environment (WF) reduces the damage of chronic stress to liver, and excitability of neurons. (A) HE staining of the liver tissues in all the groups (100×). NC represents the normal control group. CM represents the chronic stress model group. WF represents the waterfall forest environment group. NR represents the natural restoration group. (B) Effects of WF on the level of adrenocorticotropic hormone (ACTH) in all the groups. (C) Effects of WF on the level of cortisol (CORT) in all the groups. *p < 0.05 and **p < 0.01 vs. the normal control (NC) group. #p < 0.05 and ##p < 0.01 vs. the chronic stress model (CM) group. [file Image_1.TIF]

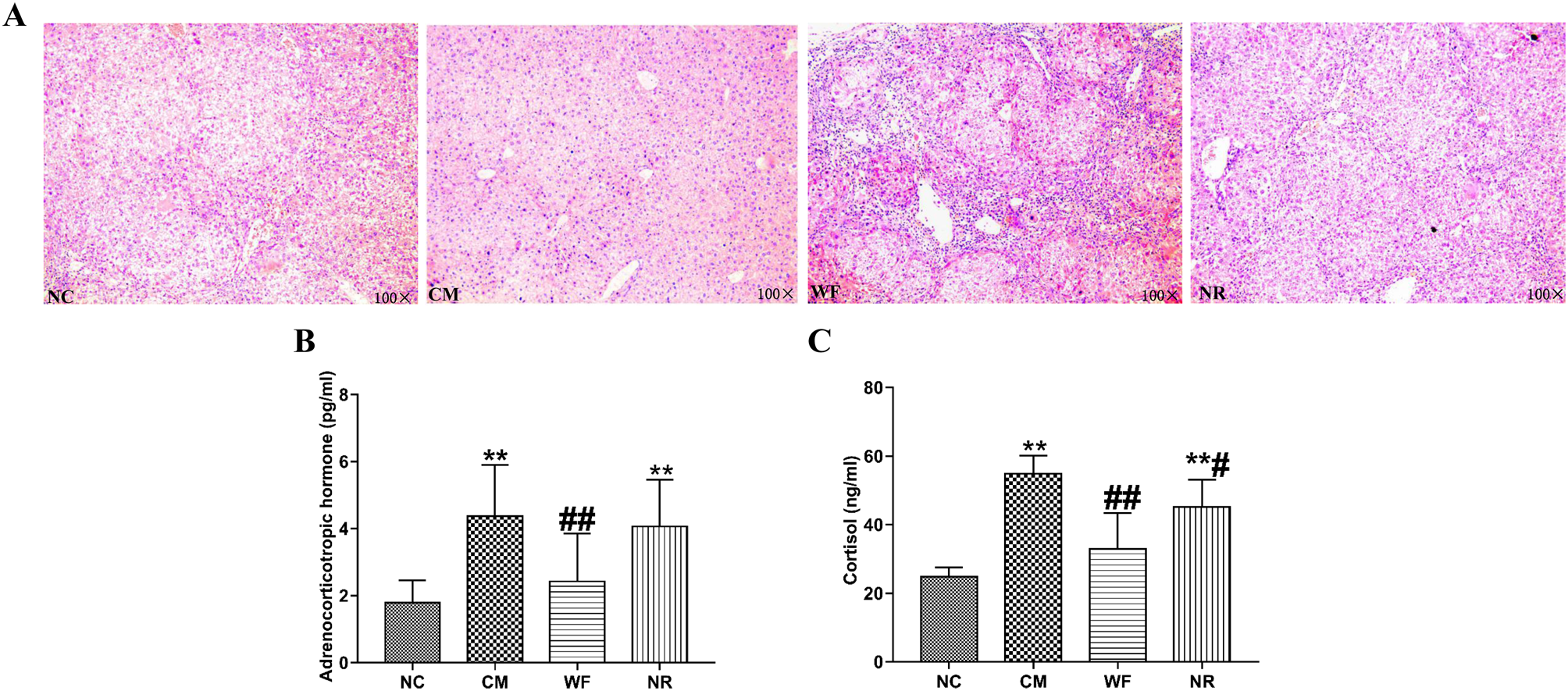

Supplement: Supplementary Figure 2 — The waterfall forest environment inhibited chronic stress by regulating the NOX4/ROS/NF-κB signaling pathway. [file Image_2.TIF]
