# Supplementary material for: Waterfall Forest Environment Regulates Chronic Stress via the NOX4/ROS/NF-κB Signaling Pathway
Source: Front Neurol. 2021 Mar 18;12:619728. doi: 10.3389/fneur.2021.619728 (PMC8044934; doi:10.3389/fneur.2021.619728)
Supplement: Supplementary file 5 [file Data_Sheet_1.docx]

**Supplementary methods**

**Psychological evaluation**

Psychological questionnaires were performed before and after the waterfall forest or urban programs. The Hamilton Anxiety Scale (HAMA) and Hamilton Depression Scale (HAMD) were used to evaluate anxiety and depression and their severity for all the participants. The HAMA included 14 items, all of which were scored from 0 (not present) to 4 (severe). The HAMD included 17 items. Among these items, 8 items were scored from 0 (not present) to 4 (severe) and 9 items were scored from 0 (none) to 2 (symptom-specific severity descriptor). The Fatigue Scale-14 (FS-14) was used to evaluate fatigue severity. The scale contained 14 items (physical fatigue, 8 items; mental fatigue, 6 items). Each item was measured with score 0-1. Higher scores indicate greater fatigue severity.

**Cognitive function measurement**

Stroop test: It consists of three parts: word test, color test and color word interference test. Word test: Participants were asked to read as many black and white printed words with red, green and blue characters as possible within 45 s. Color test: Participants were asked to read as many colors with 3 symbols (red, green and blue) as possible within 45 s. Color word interference test: Subjects were given words printed with three colors (red, green, and blue) and asked to read as many of the colors of the words as possible, rather than the words themselves, within 45 s. The test indicator is the correct number read out for each test.

PASAT test: In the test, a series of numbers are played one after the other (with an interval of 2.4 s). Participants were asked to add the two adjacent numbers they hear and report the result.

**Behavioral experiments**

**Tail suspension test (TST)**

An enhanced immobility time was regarded as a depressive state. The mice were suspended by the tail from a ledge with adhesive tape (about 3 cm from the tip of the tail). We controlled the distance from the head of the mouse to the floor was approximately 5 cm. During the 5-min test procedure, each mouse was permitted to adapt for 2 min after being suspended, a stopwatch was used to record the amount of time each mouse still stayed during the remaining 4 min.

**Morris water maze test (MWM)**

The MWM test were performed in a circular pool with a diameter of 1.5 m and 50 cm height with 22°C filled water to 40 cm deep. Escape latency was monitored using the SMART-LD program. The training schedule was composed of two trials per day more than 4 days of testing, and we determined the ability of each mouse to reach the platform within 60s. On day 5, the platform was removed and the mice were required to swim for 60s. The time spent in reaching the previous platform location (escape latency) and the time and numbers of crossings in the destination quadrant were recorded for each mouse.
